# Regulation of muscle pyruvate dehydrogenase activity and fuel use during exercise in high-altitude deer mice

**DOI:** 10.1242/jeb.246890

**Published:** 2024-08-20

**Authors:** Soren Z. Coulson, Sulayman A. Lyons, Cayleih E. Robertson, Benjamin Fabello, Lauren M. Dessureault, Grant B. McClelland

**Affiliations:** Department of Biology, McMaster University, 1280 Main Street West, Hamilton, ON, Canada, L8S 4K1

**Keywords:** Hypoxia, Running, *Peromyscus maniculatus*, Acclimation, Adaptation, Metabolic regulation, Phenotypic plasticity, Lactate

## Abstract

Adult, lab-reared, highland deer mice acclimate to hypoxia by increasing reliance on carbohydrates to fuel exercise. Yet neither the underlying mechanisms for this shift in fuel use nor the impact of lifetime hypoxia exposure experienced in high alpine conditions, are fully understood. Thus, we assessed the use of fuel during exercise in wild highland deer mice running in their native environment. We examined a key step in muscle carbohydrate oxidation – the regulation of pyruvate dehydrogenase (PDH) – during exercise at altitude in wild highlanders and in first generation (G_1_) lab-born and -raised highlanders acclimated to normoxia or hypoxia. PDH activity was also determined in the gastrocnemius of G_1_ highlanders using an *in situ* muscle preparation. We found that wild highlanders had a high reliance on carbohydrates while running in their native environment, consistent with data from hypoxia-acclimated G_1_ highlanders. PDH activity in the gastrocnemius was similar post exercise between G_1_ and wild highlanders. However, when the gastrocnemius was stimulated at a light work rate *in situ*, PDH activity was higher in hypoxia-acclimated G_1_ highlanders and was associated with lower intramuscular lactate levels. These findings were supported by lower PDH kinase 2 protein production in hypoxia-acclimated G_1_ mice. Our findings indicate that adult phenotypic plasticity in response to low oxygen is sufficient to increase carbohydrate reliance during exercise in highland deer mice. Additionally, variation in PDH regulation with hypoxia acclimation contributes to shifts in whole-animal patterns of fuel use and is likely to improve exercise performance via elevated energy yield per mole of O_2_.

## Teaser

**Summary:** High-altitude deer mice increase exercise carbohydrate use with adult hypoxia acclimation by altering pyruvate dehydrogenase regulation.

## INTRODUCTION

The ability of mammals to effectively exercise in the low oxygen conditions of high altitude has fascinated researchers for decades (e.g. Hall et al., 1936). Much of the energetic requirements of endurance exercise are met using aerobic metabolism; however, O_2_ availability is limited at high elevations. While evolved changes of the O_2_ transduction cascade in highland native species have received some attention (Ivy and Scott, 2015), data on substrates (fuels) used to power locomotion at high altitude has been somewhat equivocal, especially for lowlanders (e.g. Griffiths et al., 2019). We have previously shown that highland mice, both Rocky Mountain deer mice (*Peromyscus maniculatus*) and Andean leaf-eared mice (*Phyllotis* sp.) have a greater reliance on carbohydrate oxidation during submaximal exercise, when compared with lowland conspecifics and congenerics, respectively (Lau et al., 2017; Schippers et al., 2012, 2021). A greater reliance on carbohydrates during exercise may provide an adaptive advantage in low O_2_ conditions because of the superior yield of ATP per mole O_2_ consumed compared with fatty acid oxidation. However, the underlying mechanisms responsible for this shift in fuel use remain unclear. Moreover, evidence from research on lab-born and -raised highland deer mice suggests this fuel use strategy is the result of an evolved phenotypic plasticity in response to chronic hypoxia as adults. In first generation (G_1_) lab-born and -raised highland and lowland deer mice acclimated to normoxia, fuel use is similar during submaximal exercise. In contrast, acclimation to hypobaric hypoxia results in an increased reliance on carbohydrate oxidation to power exercise, but only in highland deer mice (Lau et al., 2017). These G_1_ deer mice were born and raised at low altitude and may not express the same phenotype as wild highland mice exposed to hypoxia throughout their life cycle, including periods where developmental plasticity may occur (Hammond et al., 2002; Robertson and Wilsterman, 2020). Indeed, exercise fuel use has never been qualified in wild, highland native mammals in their natural environment. As a result, the impact of exposure to high-altitude conditions from conception to adulthood on exercise metabolism is currently unclear.

Many aspects of muscle phenotype impact whole-animal exercise metabolism; however, in adult G_1_ highland deer mice, many of these traits are resistant to hypoxia acclimation. For example, the fibre type composition and capillarity of gastrocnemius muscle does not change with hypoxia acclimation (Lui et al., 2015), nor do these traits differ from wild highlanders (Scott et al., 2015). So, changes in whole-animal exercise metabolism with hypoxia acclimation are likely to be associated with changes in muscle fibre metabolic machinery. Variation in muscle carbohydrate use during exercise can occur because of changes in hierarchical and/or metabolic regulation of steps in the carbohydrate oxidative pathway (i.e. involving changes in protein concentration or without changes in concentration, respectively; Suarez and Moyes, 2012). Previous work has shown that hypoxia acclimation increases the apparent maximal activity (*V*_max_) of the enzyme hexokinase in the gastrocnemius muscle of G_1_ highlanders (Lau et al., 2017). Since changes in activity of this enzyme significantly affect muscle glucose uptake in exercising lab mice (Fueger et al., 2004a,b), hypoxia-acclimated highland deer mice may have an increased capacity for muscle glucose uptake. However, activities of other key glycolytic enzymes, mitochondrial volume density and mitochondrial respiratory capacity were all unchanged in the gastrocnemius of highland mice after hypoxia acclimation (Lui et al., 2015; Lau et al., 2017; Mahalingam et al., 2017), suggesting that hierarchical regulation is generally not responsible for shifts in fuel use. Instead, highlanders probably rely on regulatory changes to the carbohydrate oxidation pathway during periods of low O_2_ availability that shift fuel use patterns.

Unlike hierarchical regulation, changes in muscle metabolic regulation in response to hypoxia acclimation are poorly understood. One potential site of regulation is the irreversible conversion of pyruvate to acetyl-CoA in a series of reactions catalysed by the pyruvate dehydrogenase (PDH) complex, a key regulatory step in carbohydrate oxidation. Variation in the regulation of this reaction can significantly impact muscle carbohydrate use, specifically the proportion of glucose that is ultimately oxidized at the mitochondria (Small et al., 2018). PDH activity is covalently regulated by the relative activities of PDH phosphatase and PDH kinase (PDK), where PDH is inhibited by phosphorylation and activated in the dephosphorylated state (Holness and Sugden, 2003). Elevation of PDH activity (PDHa) could explain the high carbohydrate reliance during submaximal exercise in hypoxia-acclimated G_1_ highlanders. Whether wild highlanders or hypoxia-acclimated G_1_ highlanders have elevated PDHa during submaximal exercise is unknown. An increase in PDHa could be accomplished by at least two non-mutually exclusive mechanisms: an increase in total (i.e. maximal) PDH activity (PDHt) or a decreased capacity for PDH phosphorylation.

We studied wild highland native deer mice running at high altitude and determined their fuel use during submaximal exercise. We tested the hypothesis that lifetime exposure to low O_2_ increases exercise carbohydrate use and predicted that wild highland deer mice exercising at high altitude would show a greater reliance on carbohydrate oxidation than reported for normoxia- and hypoxia-acclimated G_1_ highland mice. We further hypothesised that the hypoxia acclimation-induced increase in carbohydrate use during submaximal exercise in G_1_ highland deer mice is associated with increased PDH activity. Therefore, we predicted that hypoxia-acclimated G_1_ highland deer mice would have higher PDHa compared with normoxia-acclimated G_1_ highlanders following submaximal muscle stimulation or running. We further predicted that increases in PDHt and/or decreases in PDK abundance would explain the hypoxia acclimation-induced increase in PDHa. To test this hypothesis, we examined the effects of hypoxia acclimation on PDH activation during submaximal muscle activation in highland deer mice. We measured PDHa in locomotory muscle following submaximal *in situ* stimulation and after submaximal running. In addition, we determined the activity of PDHt and the abundance of PDK and PDH phosphatase (PDP)1 as possible explanations for any variation in PDHa.

## MATERIALS AND METHODS

All procedures were approved by the McMaster University Animal Research Ethics Board in accordance with guidelines from the Canadian Council on Animal Care, and the Colorado Department of Fish and Wildlife.

### Running at high altitude

Adult deer mice (*Peromyscus maniculatus rufinus*) were trapped at the summit of Mt Blue Sky (Clear Creek County, CO; 39°35′18″ N, 105°38′38″ W; 4350 m a.s.l.) as described previously (Schweizer et al., 2019). Mice were kept at the summit in clear plastic disposable cages (Lab Supply, TX) with water and food (apple slices, oats and peanut butter) available *ad libitum*. At the summit (12.7±0.04 kPa O_2_, 11.3±0.3°C), running-induced maximal oxygen consumption (*V̇*_O_2_,max_) was determined using a motorized treadmill as previously described for mice (Schippers et al., 2012; Templeman et al., 2012; Lui et al., 2015; Lau et al., 2017). Briefly, mice were placed on a rodent treadmill enclosed in a metabolic chamber (volume ∼800 ml) and allowed to adjust for several minutes. Flow-through respirometry (FoxBox, Sable Systems, NV) was used to determine *V̇*_O_2__ and carbon dioxide production (*V̇*_CO_2__) as the mice rested quietly on the treadmill (Schippers et al., 2012, 2021). The treadmill was then started at 7 m min^−1^ and speed was increased by 3 m min^−1^ every 2 min until *V̇*_O_2_,max_ was reached following criteria previously established for deer mice (Lui et al., 2015; Lau et al., 2017). At least 48 h after *V̇*_O_2_,max_ was determined, mice were fasted for 3 h before running for a total of 15 min at a speed corresponding to a target intensity of 75% *V̇*_O_2_,max_ (Table 1). A second group of mice was trapped at 4350 m but was then transported to 3300 m a.s.l. for experiments in a more controlled environment (14.4±0.01 kPa O_2_, 19.7±1.0°C) using running and housing protocols as described above. *V̇*_O_2_,max_ was not available for four individuals at 4350 m and one individual at 3300 m. For these mice, an average running speed corresponding to 75% *V̇*_O_2_,max_ was determined from the *V̇*_O_2_,max_ values collected/tested from other mice at the same altitude (*N*=9 for 3300 m and *N*=7 for 4350 m).

**Table 1.**
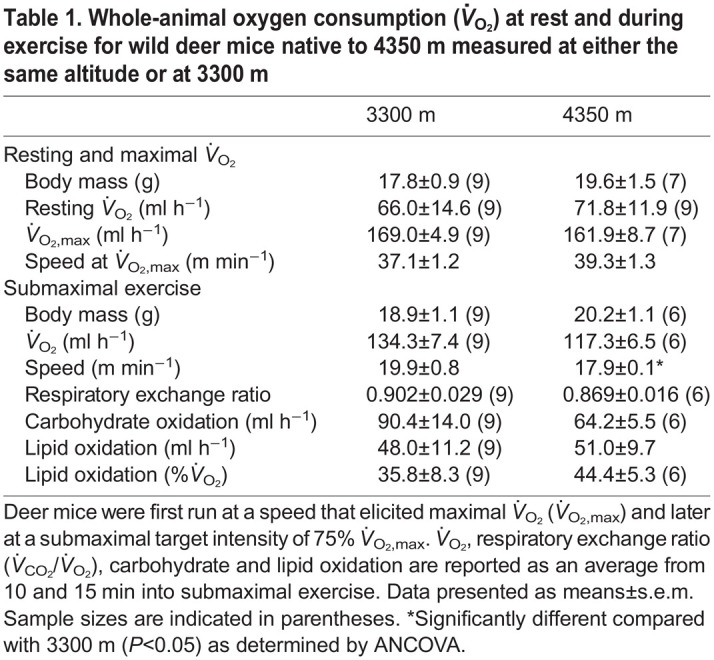
Whole-animal oxygen consumption (*V̇*_O_2__) at rest and during exercise for wild deer mice native to 4350 m measured at either the same altitude or at 3300 m

After mice ran at 4350 m, they were allowed to recover for at least 48 h with food and water available *ad libitum*, and then transported to 3300 m for tissue sampling at rest. Mice were fasted for 3–4 h, then placed in a respirometry chamber with ambient air pushed into the chamber. This air was slowly supplemented with isoflurane and once anesthetized, mice were removed from the chamber and killed by cervical dislocation and decapitation. The gastrocnemius muscle was quickly excised, flattened between two liquid-N_2_-cooled aluminium plates and placed in liquid N_2_ before transfer to −80°C. A blood sample was taken and whole-blood glucose and lactate concentrations were measured using handheld meters (Accu-Chek, Aviva, Roche Diagnostics and Lactate Pro, Arkray, Kyoto, Japan, respectively) (Lau et al., 2017). After running at 3300 m, mice were killed immediately after the submaximal running trial for post-exercise tissue sampling. Anaesthesia was induced using an isoflurane-soaked cotton ball placed in the treadmill chamber and mice were sacrificed and tissues sampled as described above.

### G_1_ highland deer mice

In a previous year, wild highland deer mice were trapped at the summit of Mt Blue Sky as described above, transported to McMaster University (<100 m a.s.l.) and bred to the first generation (G_1_). Wild breeding mice were housed in 14 h:10 h light:dark cycle, while G_1_ mice were housed in 12 h:12 h light:dark cycle after weaning. All mice were kept in normoxia at room temperature (21 kPa O_2_, ∼23°C) conditions, with food (standard rodent chow) and water *ad libitum*. G_1_ mice were at least 6 months of age before experiments began.

### Hypoxia acclimation

Adult G_1_ highland deer mice (10 females and 10 males) from 2 families were separated into one of two environmental conditions, normoxia in a temperature controlled rodent incubator (23°C, 21 kPa O_2_; Powers Scientific, Inc) or in hypoxic conditions (22-23°C, 12 kPa O_2_) using hypobaric chambers (McClelland et al., 1998; Lau et al., 2017) simulating oxygen availability at high altitude. Mice undergoing hypoxia acclimation were returned to normobaria for less than 1 h per week for cage cleaning and replenishment of food and water. Acclimations lasted at least 6 weeks before any experiments began.

### Running in hypoxia and tissue sampling

Running experiments were performed in the G_1_ mice using methods as described above, with modifications. For these experiments, dry, CO_2_-free air was mixed with N_2_ to replicate O_2_ availability at 4350 m via normobaric hypoxia (12% O_2_) and ‘pushed’ into an 800 ml Plexiglas-enclosed motorized treadmill at a rate of 1.25 l min^−1^, controlled by a mass flow controller (Sable Systems, NV). The treadmill was started at an initial speed of 7 m min^−1^ and increased by 3 m min^−1^ every 2 min until the mice reached the desired speed to elicit 75% *V̇*_O_2_,max_ determined previously for G_1_ highlanders (23.4 and 20.3 m min^−1^ for normoxia and hypoxia acclimated highlanders, respectively; Lau et al., 2017). Mice were then run for ∼15 min and then killed post exercise for tissue sampling as described above.

### *In situ* gastrocnemius stimulation

In a separate group of G_1_ highlanders, and following normoxia or hypoxia acclimation, mice were fasted for 2–4 h before direct stimulation of the gastrocnemius using an *in situ* muscle preparation. Procedures for dissection and setup of *in situ* stimulation equipment were similar to previous protocols (MacIntosh et al., 2011; Wilson et al., 1967), with modifications. Mice were placed under general anaesthesia using 3% isoflurane, and anaesthesia was confirmed by the loss of the toe pinch withdrawal reflex. Isoflurane was then reduced to 2%. Musculature of the right hindlimb was exposed. Incisions in the upper posterior hindlimb musculature were used to expose the sciatic nerve, which was then severed proximally to prevent input from the central nervous system. A suture was secured to the Achilles tendon, which was then severed distal to the suture and used to gently pull the gastrocnemius away from the rest of the hindlimb. The mouse was then placed on a warming pad as part of an *in situ* mouse apparatus (Aurora Scientific 809C; Aurora, ON) and isoflurane was reduced to 1–1.5% to maintain a plain of anaesthesia. The gastrocnemius suture was secured to the lever arm of a force transducer (Aurora Scientific 305C), while the rest of the hindlimb was immobilized by taping the foot and clamping the knee. Two electrodes attached to a bi-phase stimulator (Aurora Scientific 701C) were positioned on opposite sides of the exposed sciatic nerve stump innervating the gastrocnemius. We used these electrodes to induce isometric muscle contractions via bi-phase electrical pulses set to a constant current of ∼560 mA and using voltages between 0 and 80 V. Ringer's buffer (in mM: 118 NaCl, 4.7 KCl, 2.5 CaCl_2_, 1.2 KH_2_PO_4_, 0.57 MgSO_4_, 25 HEPES, 5.5 glucose, pH 7.2) warmed to 37°C was periodically applied to the muscle to prevent drying.

The gastrocnemius was stretched to an optimal length (*L*_o_) to control for the influence of muscle length on contractile properties (Rassier and MacIntosh, 2002). *L*_o_ was identified as the muscle length that produced the greatest force during a twitch contraction. Maximal force production (**F**_max_) was determined using electric stimuli of 200 Hz, at a 0.2 ms pulse width, and 0.3 s duration applied 6 times separated by 2 min of rest to limit fatigue. **F**_max_ was defined as the average difference between resting and peak tension, excluding contractions that failed to show fused tetanus. Following at least 2 min of rest, the gastrocnemius was stimulated again but at submaximal workloads standardized to **F**_max_ defined as either light (25% **F**_max_) or heavy (50% **F**_max_) workload. First, we identified the stimulation frequency necessary to elicit a given workload. Briefly, we stimulated the gastrocnemius (0.2 s delay, 0.2 ms pulse width, 0.2 s duration) 3–5 times at multiple frequencies of 30–80 Hz and selected the frequency that elicited force production closest to 25% or 50% **F**_max_. We then applied this stimulation as a train at a frequency of 0.2 trains per second over 3 min, consistent with methods used by previous studies that induced PDH activation using an *in vitro* muscle approach (Herbst et al., 2012). Immediately following the final contraction, the mouse was quickly removed and euthanized via cervical dislocation. The stimulated gastrocnemius was then removed within 15 s and freeze-clamped using aluminium tongs cooled in liquid N_2_, with the contralateral gastrocnemius frozen as a resting control (Wilson et al., 1967).

### Pyruvate dehydrogenase (PDH) activity

The activity of PDH in muscle homogenates was determined using a radiolabelled assay, as previously described (Constantin-Teodosiu et al., 1991a) with modifications (Putman et al., 1993). Frozen gastrocnemius samples were first powdered using a liquid N_2_ cooled mortar and pestle and 5–10 mg were diluted 30-fold in one of two ice-cold homogenization buffers. We used two buffers to control the phosphorylation state of PDH, via allosteric regulation of PDH phosphatase and PDK. To determine activity of the active form (PDHa), the phosphorylation state of the enzyme at the time of muscle sampling was preserved by inhibiting associated kinases and phosphatases using a buffer containing (in mM): 200 sucrose, 50 KCl, 5 MgCl_2_, 5 EGTA, 50 Tris-HCl, 50 of phosphatase inhibitor NaF, 1 DTT, 5 of kinase inhibitor dichloroacetate (DCA) and 0.1% (v/v) Triton X-100 at pH 7.8. Total PDH activity (PDHt) was determined by activation of PDH phosphatases by CaCl_2_ (Denton et al., 1972) and inhibition of PDK by DCA and depletion of endogenous ATP in a second buffer containing (in mM): 200 sucrose, 50 KCl, 5 MgCl_2_, 5 EGTA, 50 Tris-HCl, 10 glucose, 10 CaCl_2_, 1 DTT, 10 DCA, 2 U ml^−1^ hexokinase, and 0.1% Triton X-100 (v/v) at pH 7.8. Samples were then homogenized using a glass-on-glass homogenizer, followed by Teflon-on-glass homogenization (400 rpm) on ice for 30 s each. To limit vesicle formation, which masks PDH activity, homogenates were quickly snap-frozen in liquid N_2_ and stored at −80°C until further analysis.

Less than 48 h after initial freezing, homogenates (PDHa and PDHt) were thawed on ice and then vortexed immediately before use in PDH activity assays. The assay works by measuring the formation of acetyl-CoA over time, which can be attributed to the activity of dephosphorylated PDH. Assay conditions were identical between PDHa and PDHt homogenates, except that PDHt homogenates were more diluted prior to the assay (10 fold for PDHa and 20 fold for PDHt). 30 μl of each homogenate was added to 720 μl of a reaction mixture containing the PDH assay buffer (in mM), 108.3 Tris-HCl, 0.54 EDTA, 1.08 MgCl_2_, pH 7.8, with 3 NAD^+^, 1 CoASH, and 1 TPP. We assayed each sample in duplicate at 37°C using a dry block heater (Fisher Scientific, Ottawa, ON). The PDH reaction was initiated by adding 30 μl of 26 mmol l^−1^ pyruvate or 30 μl of dH_2_O as a blank. At precisely 1-, 2- and 3-min time points after adding pyruvate or dH_2_O, 200 μl of the assay mixture was removed and added to 40 μl of 0.5 N HClO_4_ to stop the reaction. We placed these acidified samples on ice for at least 5 min, after which they were neutralized with the addition of 12–14 μl of fresh 1 mol l^−1^ K_2_CO_3_. Samples were then left to sit for at least 5 min at room temperature before storage at −80°C until further analysis of acetyl-CoA.

### Acetyl-CoA assay

We determined PDH activity for PDHa and PDHt by quantifying acetyl-CoA concentrations at the three reaction time points using radiolabelled oxaloacetate as substrate, as described previously (Cederblad et al., 1990; Cooper et al., 1986) with modifications (Constantin-Teodosiu et al., 1991a). The ^14^C-oxaloacetate was prepared via the transamination of ^14^C-aspartic acid concurrently with the acetyl-CoA assay to limit spontaneous decomposition to pyruvate, using published methods (Siess et al., 1976) with modifications (Cooper et al., 1986). Briefly, we combined 9 μl of ^14^C-aspartic acid (Perkin Elmer L-^14^C(U)-Aspartic acid; 0.1 μCi μl^−1^), 5 μl of 80 mmol l^−1^ α-ketoglutarate (prepared fresh daily), 50 μl of 7 mmol l^−1^ EDTA, 20 μl of 500 mmol l^−1^ HEPES (pH 7.4), 86 μl of dH_2_O and 10 μl of 100 U ml^−1^ glutamate oxaloacetic transferase (GOT; aspartate aminotransferase). The transamination reaction was incubated at room temperature for 10 min, then was stopped with the addition of 10 μl of 1 mol l^−1^ HClO_4_ and kept on ice for 10 min. After, we neutralized the solution with 360 μl of 11 mmol l^−1^ EDTA (pH 7.4) and 40 μl of 600 mmol l^−1^ KOH, then left on ice for at least 5 min prior to use. The resulting ^14^C-oxaloacetate was used within 1 h.

Acetyl-CoA concentrations were assayed within one week of initial freezing of the PDH homogenate and within two freeze-thaw cycles. Samples were thawed and spun at 10,000 ***g*** for 3 min to pellet the insoluble fraction. The resulting supernatant was diluted with 5-40 μl dH_2_O to 200 μl, then 2 μl of 100 mmol l^−1^ DTT and 20 μl CuSO_4_-K^+^-acetate (0.429 mmol l^−1^ CuSO_4_, 114 mmol l^−1^ K^+^-acetate) were added to each sample, followed by room temperature incubation for 30 min. Samples were then incubated on ice for 10 min (Cooper et al., 1986). Roughly halfway through this incubation step, preparation of the labelled ^14^C-oxaloacetate was initiated (see above). Next, 20 μl of 60 mmol l^−1^ EDTA was added, and after a 5 min incubation at room temperature, 30 μl of 30 mmol l^−1^ NEM was added. Samples were incubated at room temperature for 5 min to remove excess CoASH. Acetyl-CoA was then converted to ^14^C-citrate by adding 10 μl of 220 U ml^−1^ citrate synthase and 20 μl of ^14^C-oxaloacetate preparation to each sample, then incubated at room temperature for 20 min. After the reaction was complete, excess ^14^C-oxaloacetate was converted to ^14^C-aspartate by adding 10 μl of 100 U ml^−1^ GOT and 20 μl of 275 mmol l^−1^ glutamate was added to each sample, then incubated at room temperature for 20 min. ^14^C-aspartate was then removed with the addition of 1 ml of 37.5% (w/v) Dowex cation exchange resin slurry (5OW-X8 H^+^ form, 100-200 mesh) and mixed via inversion for 2 min. Samples were centrifuged at 1200 ***g*** for 5 min to sediment the resin and 500 μl supernatant was added to 5 ml scintillation cocktail (EcoScint A; National Diagnostics, Atlanta, GA) in 20 ml scintillation vials (Wheaton; Millville, NJ). We quantified ^14^C-citrate using liquid scintillation counting (Perkin-Elmer; Waltham, MA) and acetyl-CoA concentrations were calculated using a standard curve unique to each ^14^C-oxaloacetate preparation. PDH activity (either PDHa or PDHt) was then calculated as the average rate of acetyl-CoA formation between duplicates and corrected for background activity recorded in blank runs.

### Enzyme assays

The apparent *V*_max_ of lactate dehydrogenase (LDH), pyruvate kinase (PK), citrate synthase (CS), β-hydroxyacyl-CoA dehydrogenase (HOAD), and malate dehydrogenase (MDH) were assayed *in vitro* as described previously for deer mice (Lui et al., 2015; Lau et al., 2017; Dawson et al., 2016). Frozen gastrocnemius samples were powdered using a liquid N_2_ cooled mortar and pestle, and 5–10 mg of muscle was diluted 20-fold and homogenized on ice in a buffer containing (in mM): 100 KH_2_PO_4_, 5 EDTA, 0.1% v/v Triton X-100 using a glass-on-glass homogenizer. Measurements were performed in triplicate at 37°C in 96-well format (SpectraMax Plus 384 Plate Reader, Molecular Devices, CA) with a blank lacking substrate to determine background activity. Assay conditions were as follows (in mM) for LDH: 0.28 NADH, 40 Tris-HCl (pH 7.4),±1 pyruvate; CS: 40 Tris-HCl (pH 8.0), 0.1 DTNB, 0.22 acetyl-CoA±0.5 oxaloacetate; PK: 50 imidazole, 5 ADP, 100 KCl, 10 MgCl_2_, 0.15 NADH, 10 fructose-1-6-P, 9.25 U well^−1^ LDH±5 PEP; HOAD: 100 TEA-HCl (pH 7.0), 0.15 NADH±0.1 acetoacetyl-CoA; MDH: 100 KH_2_PO_4_ (pH 7.4), 0.15 NADH±0.5 oxaloacetate. Absorbance was measured at 340 nm for all assays except CS, which was assayed at 412 nm.

### Muscle lactate

Muscles sampled during *in situ* stimulation experiments were assayed for intramuscular lactate concentration. Frozen gastrocnemius was homogenized in 10 volumes of 6% HClO_4_ on ice, then centrifuged at 10,000 ***g*** for 10 min at 4°C. The supernatant was neutralized with 3 mol l^−1^ K_2_CO_3_. The homogenate was spun again, and the supernatant was taken to measure lactate as described previously (Le Moine et al., 2011). Briefly, muscle homogenate was added to buffer (0.2 mol l^−1^ glycine buffer, 600 mmol l^−1^ glycine and 500 mmol l^−1^ hydrazine, 2.56 mmol l^−1^ NAD^+^ in dH_2_O) and absorbance was measured before and after incubation with LDH (8 U well^−1^) for 30 min at 37°C in 96-well format as described above.

### Western blot analysis

We assessed the protein levels of muscle PDH kinase isoforms (PDK1, PDK2, PDK4) and a phosphatase (PDP1) in the left gastrocnemius via western blot analysis as previously described for deer mice (Robertson et al., 2019). As a mitochondrial enzyme, whole-tissue PDK abundance varies with mitochondrial content, so the level of citrate synthase (CS) was also measured to control for variation in mitochondrial abundance. Although other indices of mitochondrial content have been identified (Larsen et al., 2012), CS is closely associated with mitochondrial density, as determined by electron microscopy (EM) in humans (Larsen et al., 2012) and rabbit muscle (Reichmann et al., 1985). Moreover, transmission EM of G_1_ highland deer mice gastrocnemius muscle shows no significant effect of hypoxia acclimation on mitochondrial density, consistent with data using CS activity (Lui et al., 2015). Each muscle sample was powdered using a liquid N_2_ cooled mortar and pestle, then homogenized using a motorized homogenizer in ice cold Radio-Immunoprecipitation Assay (RIPA) lysis buffer containing (in mM), 150 NaCl, 50 Tris-HCl, 1.0% Triton X-100, 0.5% deoxycholic acid and 0.1% sodium dodcecyl sulfate (SDS) at pH 8.0. Total protein concentration of these homogenates was determined using a Bradford assay (BioRad). Denaturation of the homogenate was then carried out in a Laemmli buffer for 5 min at 100°C. Proteins were separated on a 12% SDS-polyacrylamide gels (BioRad) for ∼30 min at 100 V and then for 1 h at 150 V in a Mini-Protein Tetra System (BioRad). The proteins were then transferred to polyvinylidene difluoride membranes (BioRad) at 25 V for 7 min using a Trans Blot Turbo Transfer System (BioRad). Membranes were incubated in blocking solution (5% skimmed milk powder in PBS-Tween (PBST) buffer (in mM), 1.5 NaH_2_PO_4_·H_2_O, 8.1 Na_2_HPO_4_, 145.5 NaCl, 0.05% Tween-20, at pH 7.4) overnight at 4°C. The membranes were then briefly washed 3 times with PBST buffer at room temperature, then incubated for 1 h with primary antibody for PDK1 (1:2000 dilution of rabbit monoclonal; Abcam, ab202468), PDK2 (1:500 dilution of rabbit polyclonal; ThermoFisher Scientific, 15647-1-AP), PDK4 (1:2000 dilution of rabbit polyclonal; ThermoFisher, 12949-1-AP), PDP1 (1:1000 dilution of rabbit monoclonal; New England Biolabs, D8Y6 L) or CS (1:1000 dilution of rabbit monoclonal; Abcam, ab129095) in blocking solution. Membranes were briefly washed again 3 times with PBST buffer, then incubated for 1 h at room temperature with secondary goat anti-rabbit IgG antibody (for PDK1, PDK2 and PDK4, and PDP1; Invitrogen, 31466 diluted at 1:5000), or donkey anti-rabbit antibody (for CS; Santa Cruz Biotechnology diluted at 1:10,000). Membranes were developed in an ECL clarity solution (BioRad), and the bands were imaged using a ChemiDoc MP Imaging system (BioRad). Each gel contained one common sample to standardize between gels, and the software ImageLab^TM^ (BioRad) was used to quantify the intensity of the bands. Following this step, each membrane was stained with Coomassie Blue (BioRad) to obtain total loaded protein in each well, which was used to correct for variation in relative protein abundance for each sample.

### Calculations and statistics

Resting and exercise *V̇*_O_2__ and *V̇*_CO_2__ were calculated using equation 3b from Withers (1977) and fuel oxidation during submaximal running calculated as outlined in Frayn (1983). Any negative rates of substrate oxidation were assumed to be 0. This occurred for lipid oxidation in one individual running at 3300 m. Fuel use is also presented as a percentage of total *V̇*_O_2__. For exercise metabolism, we found no significant differences in running data between 10 and 15 min. Thus, these time points were combined for each individual mouse.

Comparisons between groups were performed using an ANCOVA with body mass as a covariate (wild mice *V̇*_O_2__, carbohydrate and lipid oxidation at 3300 m and 4350 m) in R (http://www.r-project.org), an unpaired *t*-test (respiratory exchange ratio, western blot), a one-way ANOVA (enzyme activities between G_1_ highlanders and wild highlanders) or two-way ANOVA with workload and acclimation condition as main effects (*in situ* PDH and lactate) in Graphpad Prism (version 10.0.0) for Mac (GraphPad Software, Boston, MA, USA). Outliers were identified using a Grubb's test and removed. All tests used an alpha value of 0.05.

## RESULTS

### Exercise performance at high altitude

**Fig. 1.**
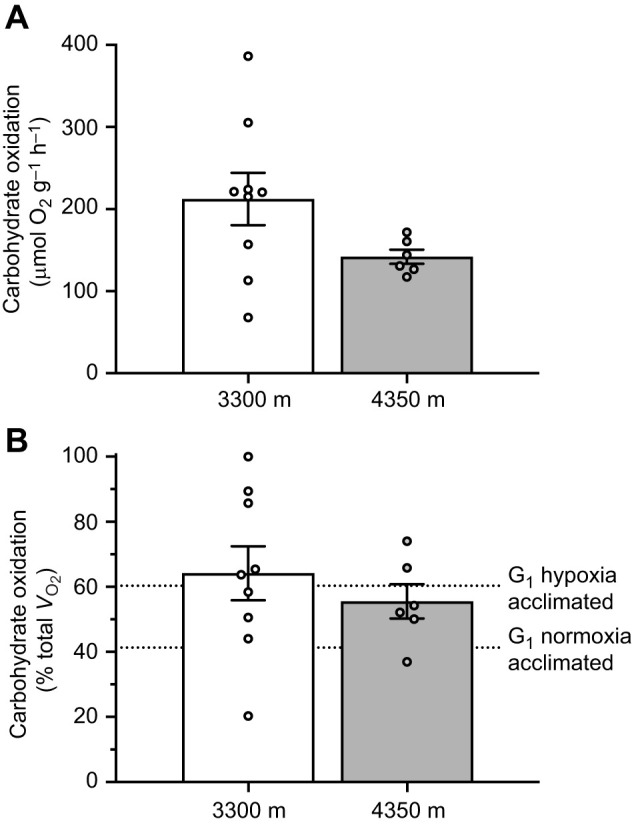
**Carbohydrate use in wild highland deer mice native to 4350 m during submaximal running exercise at two test altitudes.** Mice were exercised at 75% *V̇*_O_2_,max_ at 3300 m a.s.l. (*n*=9) or 71% *V̇*_O_2_,max_ at 4350 m (*n*=6). Carbohydrate oxidation rates calculated using respirometry and standardized to body mass (A) or to total O_2_ consumption (*V̇*_O_2__) (B). Absolute carbohydrate oxidation rates were not significantly different in mice running at 4350 m or at 3300 m (*F*_1,12_=2.863, *P*=0.116, mass included as a covariate). When carbohydrate use was expressed as a percentage of total oxygen consumption there was no significant difference between altitudes (*t*_13_=0.778, *P*=0.450). Dotted lines indicate previously published average values for first generation (G_1_) lab-born and -raised highland deer mice acclimated to normoxia or hypoxia (Lau et al., 2017) exercising at 75% *V̇*_O_2_,max_ in 12% O_2_. Data presented as means±s.e.m.

**Fig. 2.**
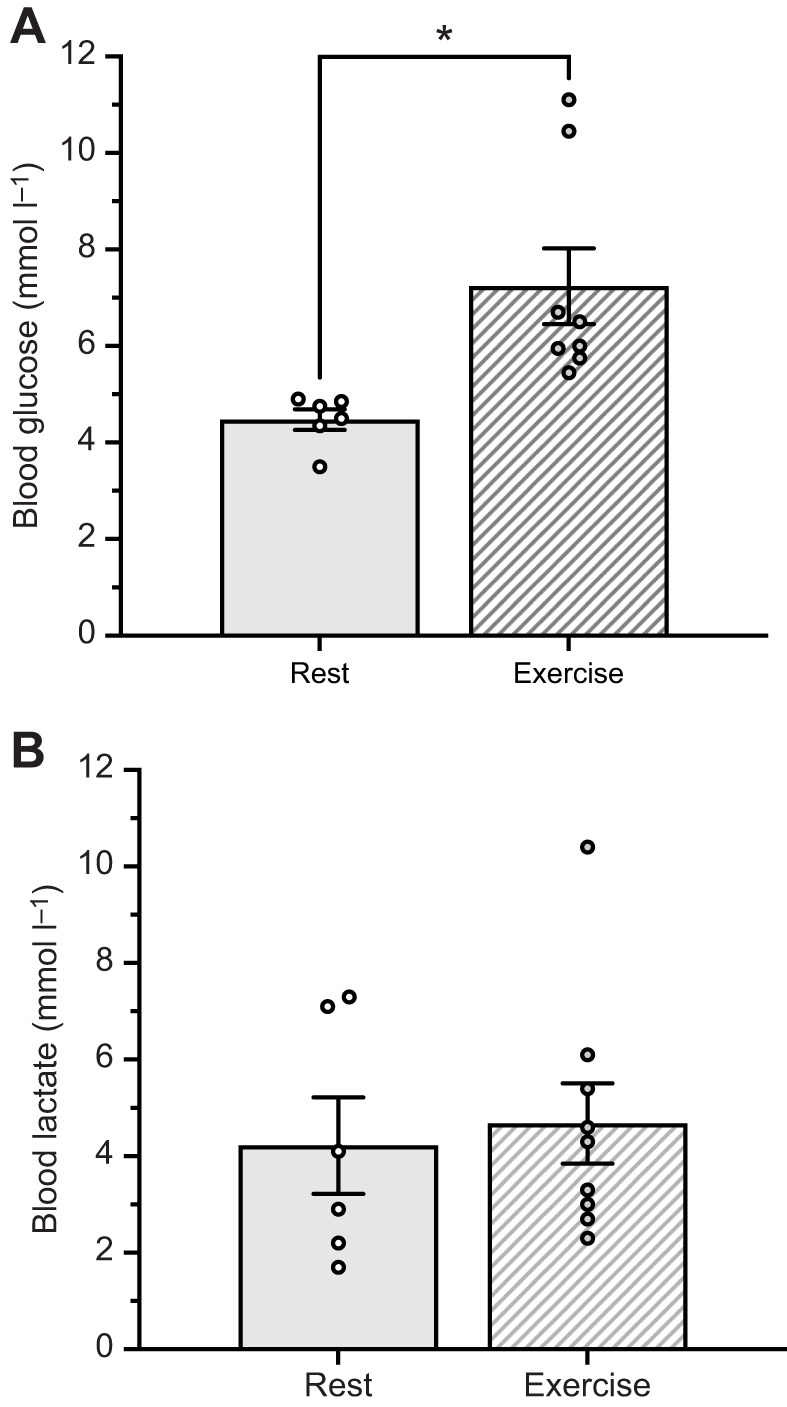
**Whole-blood concentrations of glucose and lactate in wild highland deer mice native to 4350 m at rest and following 15 min exercise at 75% *V̇*_O_2_,max_ at an altitude of 3300 m a.s.l.** (A) Blood glucose and (B) blood lactate concentrations. *N*=6 and *N*=8 for glucose and lactate at rest and exercise, respectively. *, indicates significant difference between rest and post-exercise (*P*<0.05). Data presented as means±s.e.m.

To understand whole-animal exercise performance at high altitude, wild highland deer mice were run at their native altitude of 4350 m (12.7±0.04 kPa O_2_, at an ambient temperature of 11.3±0.3°C), with a separate group of highland mice run in a more controlled lab setting at 3300 m (14.4±0.01 kPa O_2_, at an ambient temperature of 19.7±1.0°C). Since exercise intensity relative to maximal aerobic capacity determines the proportional use of carbohydrates and lipids (Schippers et al., 2014), we first measured running *V̇*_O_2_,max_ to standardize submaximal intensities. We found that *V̇*_O_2_,max_ did not differ between testing altitudes (*F*_1,13_=2.243, *P*=0.158), nor did the average speed at which highland mice reached *V̇*_O_2_,max_ (*P*>0.05) or 75% *V̇*_O_2_,max_, although it approached significance (*P*=0.050; Table 1). Similarly, there were no significant differences in running *V̇*_O_2__, respiratory exchange ratio, or lipid and carbohydrate oxidation between testing altitudes (Table 1). Both mass-specific carbohydrate oxidation rates and carbohydrate oxidation rates relative to *V̇*_O_2__ were also similar between testing environments (*P*>0.05; Fig. 1). The carbohydrate oxidation values in wild highland deer mice were in line with fuel use patterns previously observed for G_1_ highland deer mice after hypoxia acclimation (indicated by dotted lines in Fig. 1B; Lau et al., 2017). Immediately after running for 15 min at 3300 m, blood glucose increased 1.6× from values at rest (*P*=0.012), whereas blood lactate remained unchanged (*P*>0.05; Fig. 2).

### Total PDH and PDH activity for *in situ* stimulated gastrocnemius muscle of G_1_ mice

**Fig. 3.**
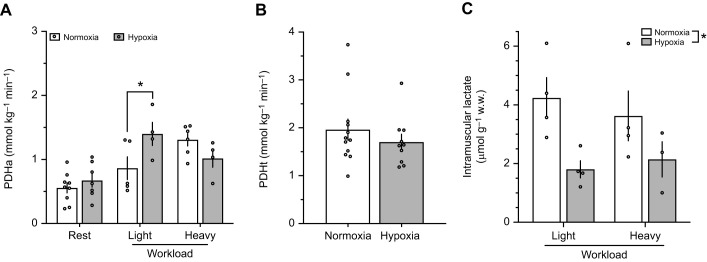
**Pyruvate dehydrogenase activity and lactate accumulation in isolated gastrocnemius muscle at rest or stimulated to contract in an *in situ* muscle preparation, in first generation lab-born and -raised highland deer mice acclimated to normoxia (21 kPa O_2_) or hypobaric hypoxia (12 kPa O_2_).** (A) Pyruvate dehydrogenase activity (PDHa), (B) intramuscular lactate accumulation and (C) total PDH (PDHt) activity. The gastrocnemius was stimulated to contract at one of two submaximal workloads standardized to maximal force production (**F**_max_): 22.1±4.0% and 50.7±1.9% **F**_max_ for light and heavy workloads, respectively, in normoxia-acclimated mice and 22.2±1.1% and 45.1±1.6% **F**_max_, respectively, for hypoxia-acclimated mice. The contralateral gastrocnemius was not stimulated and was taken as a resting control. PDH activity standardized to muscle wet mass and measured with *in vivo* phosphorylation state preserved (PDHa) and following dephosphorylation for total (i.e. maximal) activity (PDHt). For PDHa there was a significant main effect of workload (*F*_2,29_=15.59, *P*<0.0001) and a significant workload×acclimation interaction (*F*_2,29_=4.703, *P*=0.017). Intramuscular lactate showed a significant main effect of acclimation (*F_1,11_=8.801, *P*=0.0109, light and heavy workload data combined). Sample sizes for PDHa were *N*=9 and *N*=7 at rest, *N*=5 and *N*=4 for light workload and *N*=6 and *N*=4 for heavy workload for normoxia and hypoxia acclimated mice, respectively. For lactate *N*=4 and *N*=4 for light workload, and *N*=4 and *N*=3 for heavy workload, for normoxia and hypoxia acclimated mice, respectively. For PDHt, *N*=12 and *N*=10 for normoxia and hypoxia acclimated mice, respectively. Data presented as means±s.e.m.

Whole-animal fuel use during exercise is primarily the result of substrate oxidation in skeletal muscles recruited for locomotion. Therefore, we examined metabolic traits of working gastrocnemius muscle from normoxia and hypoxia acclimated G_1_ highland deer mice. We determined the activity of PDH, where phosphorylation state was preserved from *in vivo* (PDHa) and total (i.e. maximal) PDH activity, where PDH was dephosphorylated (PDHt). To study contracting gastrocnemius under physiological conditions, precisely control muscle work rate, ensure adequate O_2_ delivery and rapidly sample muscle to preserve PDH phosphorylation state, we used an *in situ* muscle preparation (MacIntosh et al., 2011; McDonald et al., 1992; Segal and Faulkner, 1985). Maximal work rates (**F**_max_) of the gastrocnemius were equivalent in the two work rate groups (*F*_1,34_=2.027, *P*=0.164) and between acclimations (*F*_1,34_=1.119, *P*=0.298; Table 2). Each muscle preparation was stimulated at one of two work intensities corresponding to the equivalent of 22% (light) and 48-49% (heavy) of F_max_ for each acclimation group. Absolute work (**F**_work_) was significantly different between light and heavy work rates (*F*_1,34_=33.792, *P*<0.001) but was unaffected by acclimation (*F*_1,34_=0.5919, *P*=0.447). We found that PDHa increased from rest to immediately after stimulation (effect of workload, *F*_2,29_=14.47, *P*<0.001; Fig. 3A). There was also a significant interaction between workload and acclimation (*F*_2,29_=4.703, *P*=0.017) and in the light workload, hypoxia acclimated G_1_ highlanders had ∼60% greater PDHa compared with normoxia-acclimated mice (0.864±0.177 mmol kg^−1^ min^−1^ vs. 1.399±0.180 mmol kg^−1^ min^−1^, respectively; *P*<0.05). Since PDHt was similar between normoxia and hypoxia acclimation (1.962±0.220 mmol kg^−1^ min^−1^ and 1.655±0.154 mmol kg^−1^ min^−1^, respectively; *P*=0.371; Fig. 3B), PDHa as a percentage of PDHt increased from 45.5±11.5% in normoxia-acclimated mice to 70.0±6.4% in hypoxia-acclimated mice (*P*=0.063, one-tailed *t*-test). In contrast, at the heavy workload, PDHa was equivalent in the gastrocnemius of normoxia- and hypoxia-acclimated deer mice (1.310±0.100 mmol kg^−1^ min^−1^ and 1.017±0.138 mmol kg^−1^ min^−1^, respectively; Fig. 3A). There was no significant interaction effect between workload and acclimation for intramuscular lactate accumulation (*F*_1,11_=1.284, *P*=0.281), and we combined data from both light and heavy workloads to assess the influence of acclimation. We found a significant effect of acclimation (*F*_1,13_=8.801, *P*=0.011), which is probably driven by lower intramuscular lactate in hypoxia-acclimated highlanders (Fig. 3C).

**Table 2.**
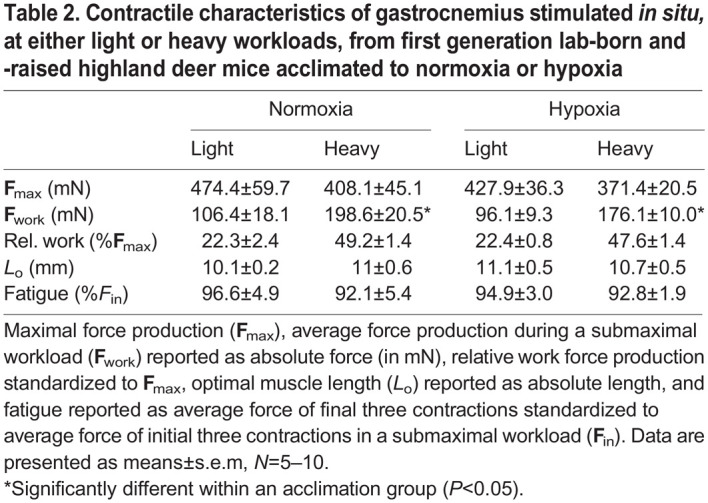
Contractile characteristics of gastrocnemius stimulated *in situ,* at either light or heavy workloads, from first generation lab-born and -raised highland deer mice acclimated to normoxia or hypoxia

### Total PDH and PDH activity post-exercise

**Fig. 4.**
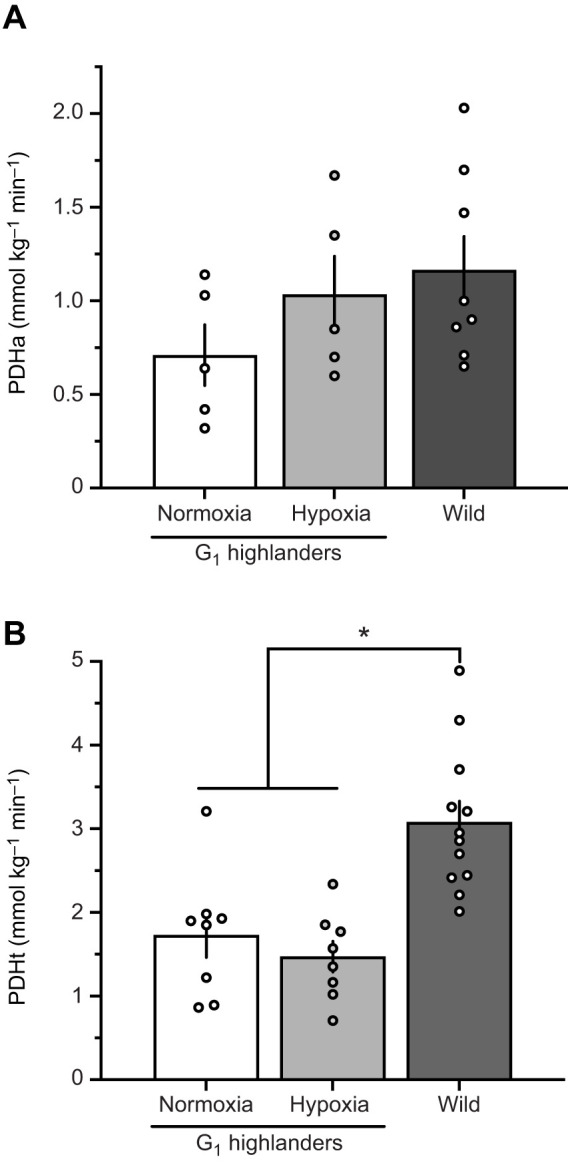
**Pyruvate dehydrogenase (PDHa) activity in the gastrocnemius muscle following submaximal running exercise (15 min at 75% *V̇*_O_2_,max_).** Both groups of first-generation lab-born and -raised (G_1_) mice were exercised in normobaric hypoxia (12% O_2_) and wild mice were exercised at an altitude of 3300 m. (A) PDH activity was standardized to muscle wet mass and measured with *in vivo* phosphorylation state preserved (PDHa) and (B) following dephosphorylation for total (i.e. maximal) activity (PDHt). There was a significant difference between the groups for PDHt (*F*_2,25_=13.53, *P*<0.001) by one-way ANOVA. *Significant difference from G_1_ highlanders from post hoc analysis (*P*<0.05). Sample sizes for PDHa were *N*=5, *N*=5 and *N*=8 for normoxia and hypoxia-acclimated G_1_ mice and wild highlanders, respectively. For PDHt *N*=8, *N*=8 and *N*=12 for normoxia and hypoxia acclimated G_1_ mice and wild highlanders, respectively. Data presented as means±s.e.m.

To test if the results from the *in situ* preparations could be recapitulated *in vivo*, PDHa and PDHt measurements were repeated in gastrocnemius muscle sampled immediately after treadmill exercise at 75% *V̇*_O_2_,max_ in G_1_ highlanders after normoxia and hypoxia acclimation. These data were compared with wild highlanders that exercised at 3300 m (Fig. 4). PDHa showed a trend to increase from normoxia-acclimated mice (0.713±0.163 mmol kg^−1^ min^−1^) to hypoxia-acclimated mice (1.032±0.205 mmol kg^−1^ min^−1^) and to wild highlanders (1.166±0.179 mmol kg^−1^ min^−1^). However, this trend failed to reach statistical significance (*F*_2,15_=1.53, *P*=0.249). For PDHt, there was a significant difference between the groups (*F*_2,25_=13.53, *P*<0.001). *Post hoc* analysis revealed that PDHt was 1.8× (*P*=0.001) and 2.1× (*P*<0.001) higher in wild highlanders compared with normoxia- and hypoxia-acclimated G_1_ highlanders, respectively (Fig. 4B).

### Muscle metabolic capacity and expression of covalent PDH modifiers following hypoxia acclimation

**Fig. 5.**
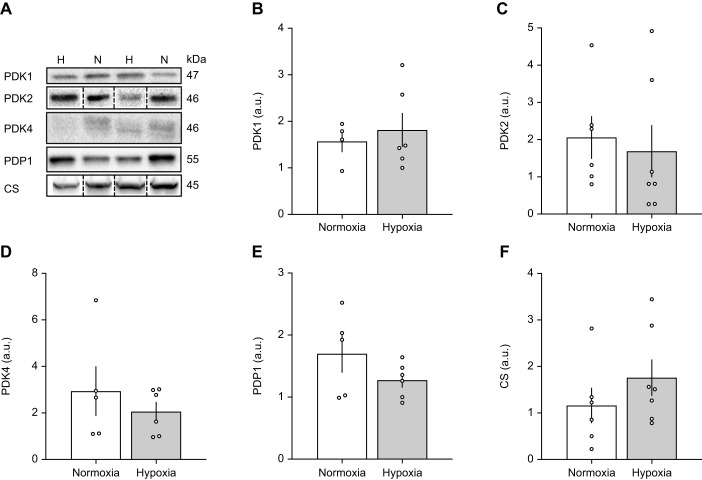
**Pyruvate dehydrogenase kinase, PDH phosphatase and citrate synthase levels in the gastrocnemius of G_1_ deer mice acclimated to normoxia or hypobaric hypoxia.** (A) Western blotting of indicated proteins from tissues collected after 15 min of exercise at 75% *V̇*_O_2_,max_ in hypoxia (12% O_2_). N, normoxia (21 kPa O_2_; representative blots of *N*=4 for PDK1, *N*=5 for PDK4 and PDP1, *N*=6 for PDK2 and CS); H, hypobaric hypoxia (12 kPa O_2_; *N*=6 for PDK1, PDK4 and PDP1, *N*=7 for PDK2 and CS). Dashed lines indicate splices where bands from PDK2 and CS were not taken from adjacent lanes. (B–F) Data presented as means±s.e.m.

**Fig. 6.**
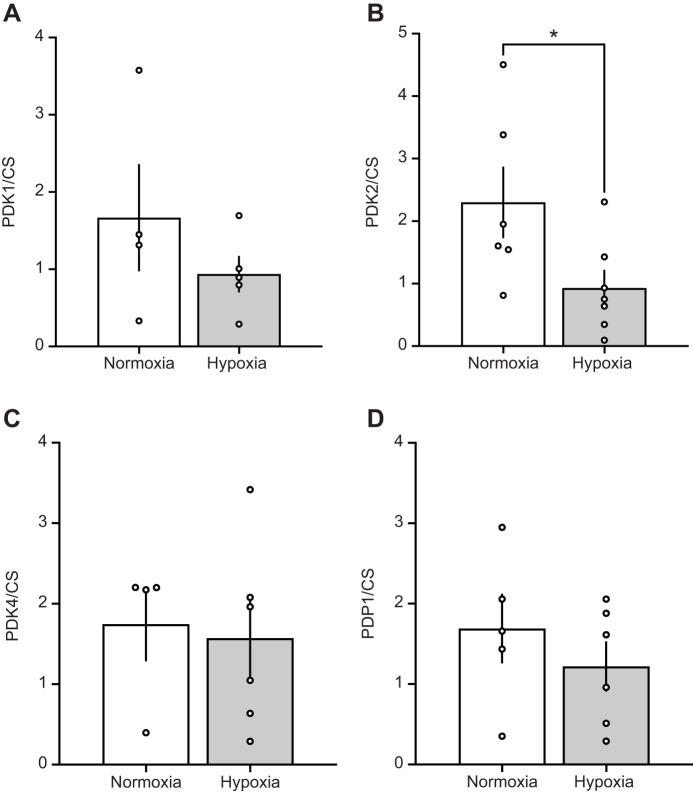
**Pyruvate dehydrogenase kinase and PDH phosphatase relative to citrate synthase protein levels in the gastrocnemius of G_1_ deer mice acclimated to normoxia or hypobaric hypoxia.** (A) PDK1, (B) PDK2, (C) PDK4 and (D) PDP1 relative to CS in mice acclimated to normoxia (21 kPa O_2_, *N*=4 for PDK1 and PDK4, *N*=5 for PDP1 and *N*=6 for PDK2) or hypobaric hypoxia (12 kPa O_2_, *N*=5 for PDK1, *N*=6 for PDK4 and PDP1, *N*=7 for PDK2). Data presented as means±s.e.m. *Significant difference between acclimations (*P*<0.05).

Covalent regulation of PDH occurs through the actions of PDK, with the isoforms PDK1, PDK2 and PDK4 produced in mammalian muscle (Gudiksen and Pilegaard, 2017; Klyuyeva et al., 2019). In the gastrocnemius, protein content of PDK1 (*P*=0.622), PDK2 (*P*=0.692) and PDK4 (*P*=0.425) were unaffected by hypoxia acclimation (Fig. 5B–D). Similarly, the protein abundance of PDP1 did not change significantly with hypoxia acclimation (Fig. 5E; *P*=0.187). CS protein content, used as a marker of muscle mitochondrial density, was 1.5× higher after hypoxia acclimation but did not reach the level of statistical significance (Fig. 5F, *P*=0.291). When protein levels were assessed relative to CS, we found that PDK1, PDK4 and PDP1 did not change significantly with hypoxia acclimation (Fig. 6). However, the abundance of PDK2 relative to CS (PDK2/CS) was 2.5× lower in hypoxia-acclimated compared with normoxia-acclimated G_1_ highland deer mice (Fig. 6B, *P*=0.043).

To further characterize the gastrocnemius phenotype, we determined the apparent *V*_max_ of five enzymes used as markers of metabolic pathway capacities. In general, *V*_max_ was similar among normoxia- and hypoxia-acclimated G_1_ highlanders and wild highlanders (*P*>0.05; Table 3). The exception was the enzyme MDH (*F*_2,21_=4.738, *P*=0.02), which was 1.7× greater (*P*=0.018) in wild highlanders compared with hypoxia-acclimated highlanders (Table 3). The *V*_max_ of the β-oxidation enzyme HOAD tended to be higher in the wild highland native mice, but this difference failed to reach statistical significance in *post hoc* analysis (*P*=0.087).

**Table 3.**
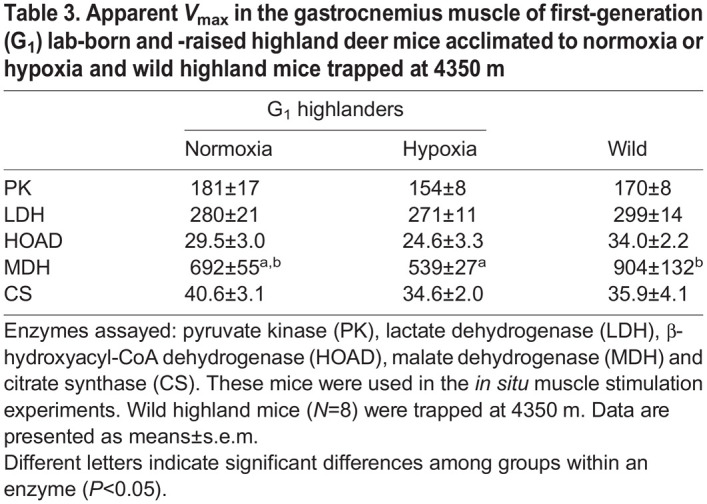
Apparent *V*_max_ in the gastrocnemius muscle of first-generation (G_1_) lab-born and -raised highland deer mice acclimated to normoxia or hypoxia and wild highland mice trapped at 4350 m

## DISCUSSION

In this study, we assessed the effects of hypoxia exposure on whole-animal carbohydrate oxidation during submaximal exercise in highland deer mice and the underlying acclimation-induced changes in muscle carbohydrate metabolism. We found that wild highland deer mice born, raised and exercised at high altitude have similar carbohydrate reliance as adult G_1_ lab-born and -raised highland deer mice acclimated to hypoxia. These data did not support our initial hypothesis that lifetime exposure to high altitude would further increase carbohydrate reliance compared with adult acclimation alone. We did find mixed support for our hypothesis of an increased PDH activation in working locomotory muscle with hypoxia acclimation. Although PDHa tended to be similar among both acclimated G_1_ and wild highlanders sampled post-running, PDHa was elevated following hypoxia acclimation when gastrocnemius muscle was stimulated directly *in situ* at a light workload. This elevation in PDHa was associated with lower muscle PDK2 protein content relative to CS after hypoxia acclimation in G_1_ mice. Elevated PDHa was also associated with lower intramuscular lactate accumulation after hypoxia acclimation, indicating greater flux of glucose through aerobic metabolic pathways, improving metabolic efficiency. Together, these results indicate that adult phenotypic plasticity is sufficient to induce whole-animal changes in exercising fuel use and that changes in metabolic regulation of carbohydrate oxidation in locomotor muscle contribute to this shift in exercise metabolism.

We show here for the first time that wild highland deer mice, running at high elevation at an intensity of 75% *V̇*_O_2_,max_, have a high reliance on carbohydrate oxidation. In fact, they use a similar mix of fuels as hypoxia-acclimated G_1_ highland mice (Fig. 1; Lau et al., 2017). These results support an important role of phenotypic plasticity in the expression of this phenotype at high altitude, with hypoxia-acclimated G_1_ highland mice and wild highland mice showing a similar exercise metabolism, which diverges from that of lowland deer mice (Lau et al., 2017). The similarity in exercise fuel use between wild highland and hypoxia-acclimated G_1_ highlanders does not provide support for developmental plasticity as a major determinant of exercise fuel use in highland deer mice. However, it is unclear if the contributions of adult and developmental phenotypic plasticity play similar roles in setting exercise fuel use patterns in other highland taxa. Wild highland leaf-eared mice from the Andes also rely to a greater extent on carbohydrate oxidation during submaximal exercise than closely related lowland mice (Schippers et al., 2012, 2021), with similar respiratory exchange ratios and relative carbohydrate use to highland deer mice. However, wild leaf-eared mice used in these studies were born and developed at high altitude but were acclimated to low-altitude conditions as adults before testing, indicating a role for developmental plasticity in exercise metabolism. Therefore, the contributions of adult and developmental plasticity in setting exercise fuel use may vary among highland taxa. Nevertheless, this common fuel use strategy has likely evolved in these groups as an oxygen saving strategy in an oxygen-limited environment.

Entry of pyruvate into mitochondria and conversion to acetyl-CoA is an important regulatory step in glucose oxidation. We found that the total capacity for pyruvate oxidation (PDHt) did not change with hypoxia acclimation in G_1_ highland deer mice. These results are consistent with data on pyruvate-fuelled mitochondrial respiration in permeabilized gastrocnemius fibres, which was unaffected by hypoxia acclimation in highland mice (Mahalingam et al., 2017). In contrast, we found hypoxia acclimation altered contraction-induced regulation of PDHa when muscle was stimulated *in situ* at a light work rate. However, we found similar PDHa among G_1_ and wild highlanders when muscles were sampled post-running (Fig. 4). This difference in results between techniques may be due to the timing of muscle sampling, which was longer *in vivo* than for *in situ* stimulation experiments, and PDH phosphorylation state can change rapidly post-exercise (Putman et al., 1995). The significant increase in PDHa at a light work rate with *in situ* stimulation (Fig. 3A) reflects an increased capacity for pyruvate oxidation after hypoxia acclimation. Greater PDHa was associated with a lower accumulation of intramuscular lactate in hypoxia-acclimated mice (Fig. 3C), despite similar LDH activity between acclimation groups (Table 3). Consistent with these results, wild highland deer mice running at altitude showed no change in blood lactate from rest to after 15 min of submaximal exercise (Fig. 2B). This suggests that hypoxia acclimation reduces reliance on anaerobic ATP production at this submaximal exercise intensity providing a metabolic efficiency advantage. Wild highlanders had significantly higher PDHt than either normoxia- or hypoxia-acclimated G_1_ highlanders. Since CS activity was similar between these groups, differences in PDHt are likely not the result of changes in mitochondrial volume (Larsen et al., 2012). The higher PDHt observed for wild highlanders (Fig. 4B) would allow for a higher scope for PDH activation compared with G_1_ highlanders. PDHa in running wild highlanders represents on average 38% of PDHt, only a modest capacity of maximal PDH activity compared with G_1_ mice, whereas PDHa was 70% of PDHt for hypoxia-acclimated highlanders.

Hypoxia acclimation in G_1_ highlanders was not associated with changes in protein levels of PDK isoforms expressed in muscle, nor for PDP1 (Fig. 5). However, hypoxia acclimation was associated with a decrease of muscle PDK2 protein abundance relative to CS (Fig. 6). Regulation of PDH in muscle is complex but a reduction in relative PDK2 protein abundance with hypoxia acclimation may lead to a reduction in total PDK activity (Dunford et al., 2011) and higher PDHa. However, the PDK1 isoform also plays an important role in the response of muscle to hypoxia. PDK1 has been linked to the O_2_-labile transcription factor, hypoxia-inducible factor (HIF)1α in cellular (Kim et al., 2006; Papandreou et al., 2006) and rodent (Dunford et al., 2011; Le Moine et al., 2011) models. While HIF-1α is elevated in acute hypoxia, levels decrease with chronic hypoxia exposure, and this is linked to an increase in PDHa in exercising CD-1 lab mice (Le Moine et al., 2011). The decrease in HIF-1α is likely mediated by cellular ‘desensitization’ to HIF by increased activity and expression of prolyl hydroxylase with chronic hypoxia (Ginouvès et al., 2008). However, our results (Figs 5 and 6) do not support decreased PDK1 expression as a mechanism for the increased PDHa seen with muscle stimulation after hypoxia acclimation (Fig. 3). Surprisingly, we found that PDHa did not increase from light to heavy workloads in hypoxia-acclimated deer mice, as previously shown in exercising humans (Constantin-Teodosiu et al., 1991b; Howlett et al., 1998). One potential explanation for this discrepancy is that the duration of electrical stimulation in our study was relatively short. In exercising humans, PDHa is similar following one minute of exercise at 65% and 90% *V̇*_O_2_,max_, but after 10 min of exercise, PDHa is higher at 90% *V̇*_O_2_,max_ than it is at 65% *V̇*_O_2_,max_ (Howlett et al., 1998). Therefore, extending our electrical stimulation may have increased PDHa in the heavy workload compared with the light workload, although it remains unclear why this does not appear to be the case for normoxia-acclimated mice. The mechanisms underlying this apparent interaction between exercise intensity and duration in determining PDHa are unclear, as are any potential interactions with hypoxia acclimation. We also found that PDHa was similar between acclimations at the heavy work rate. This is likely because PDHa approaches PDHt at high exercise intensities, which is unaffected by hypoxia acclimation. At high exercise intensities, the milieu of the mitochondrial matrix favours PDH dephosphorylation, due to Ca^2+^ accumulation, greater NAD^+^/NADH ratios and greater ADP concentrations, which collectively inhibit PDK and stimulate PDH phosphatase (reviewed by Spriet and Heigenhauser, 2002). Nevertheless, our data indicate that the kinetics of PDH activation with respect to exercise intensity are altered by hypoxia acclimation in highland deer mice, allowing a greater degree of activation at light workloads.

Muscle glucose uptake is an important determinant of exercise glucose oxidation, at least for lab mice (Wasserman et al., 2011). Wild highland deer mice showed a significant elevation in blood glucose with exercise (Fig. 2), and this would provide an increased gradient for muscle uptake. Although capacity for muscle glucose uptake is increase with hypoxia acclimation in highland deer mice, through increases in hexokinase activity (Lau et al., 2017; Fueger et al., 2004a,b), differences in glycolytic capacity do not explain acclimation effects on muscle fuel use. Hypoxia acclimation has little impact in G_1_ highlanders on the activities of other glycolytic enzymes, nor does it change mitochondrial volume density or muscle respiratory capacity (Lau et al., 2017; Lui et al., 2015; Mahalingam et al., 2017). These data suggest that hierarchical regulation of muscle metabolism is insufficient to explain the plasticity in whole-animal fuel use during exercise and that muscle metabolic regulation is likely an important mechanism involved in upregulation of glucose oxidation. For instance, muscle glucose uptake can be increased by the contraction-induced translocation of glucose transporters to the cell membrane (Jessen and Goodyear, 2005). Although hypoxia acclimation stimulates expression of *Glut4* mRNA (Lau et al., 2017), we found sarcolemmal GLUT4 protein content did not differ between normoxia- and hypoxia-acclimated G_1_ highlanders after either stimulation at light or heavy work rates (data not shown). This does not support a role for membrane glucose conductance in changes in carbohydrate use of working muscle in highland mice.

### Conclusions and perspectives

This is the first study to measure exercise fuel use in a high-altitude animal at their native elevation. We show that for highland deer mice, exercise fuel use in the wild is similar to that of lab-born and -raised highlanders acclimated to hypoxia, but not those acclimated to normoxia. Thus, there is an essential role of adult phenotypic plasticity in establishing this fuel use phenotype in highland deer mice. This is an evolved plasticity response since lowland deer mice show no change in exercise fuel use with hypoxia acclimation (Lau et al., 2017). This is consistent with data from lab rats and humans, where chronic exposure to hypobaric hypoxia did not influence exercise metabolism (McClelland et al., 1998, 2017; Griffiths et al., 2019). Previous studies have shown how highland deer mice have enhanced capacity for delivery of O_2_ (Lui et al., 2015; Tate et al., 2017, 2020) and substrates (Lyons and McClelland, 2022) to working muscle. Many of these traits are fixed at a higher level in highland compared with lowland mice. Here, we show that plasticity of metabolic regulation to affect muscle pyruvate oxidation is an important response to hypobaric hypoxia that allows highland mice to adjust exercise fuel use. This highlights the importance of metabolic regulation of muscle in the adaptation to extreme environments.
